# Stepping stones for the future—2022 major developments, journal metrics, and appreciation for reviewers

**DOI:** 10.4069/kjwhn.2022.12.14.1

**Published:** 2022-12-29

**Authors:** Sue Kim

**Affiliations:** Mo-Im Kim Nursing Research Institute, College of Nursing, Yonsei University, Seoul, Korea

The Year of the Tiger was projected to bring forth resilience and strength in the face of challenges [1]. This certainly rings true for the *Korean Journal of Women Health Nursing* (KJWHN), as we sought to follow the prior year’s success in being indexed in Scopus. I wish to describe some noteworthy accomplishments of the journal in 2022 and share some messages for potential authors.

## Major developments in 2022

On October 29, 2022, KJWHN received notice of its release to PubMed Central (PMC) live site three months after the interchange of the PMC Full-Participation Agreement on July 30, 2022 [2]. Therefore, it also became searchable in PubMed. While our September issue editorial projected that KJWHN would likely be the first non-English journal among Korean health-related journals to achieve PMC status, since its publication, the *Journal of the Korean Society of Radiology* (*Taehan Yongsang Uihakhoe Chi*) was cataloged as PubMed journal in the National Library of Medicine 14 days earlier than KJWHN. Meanwhile, KJWHN is the first non-English, non-MEDLINE nursing journal to be indexed in PMC and PubMed.

On December 5, 2022, we received official notice of being indexed in Emerging Sources Citation Index (ESCI), marking a significant milestone for KJWHN. We are delighted that published papers from 2020 (issue 1, volume 26) will be retroactively included in both PMC and Web of Science Core Collection databases in Korean and/or English. Out of more than 20 nursing journals in Korea, KJWHN is the second journal published in both Korean and English to achieve triple crown status in three databases, including PubMed, Web of Science Core Collection, and Scopus. The first one is the *Journal of Korean Academy of Nursing* (eISSN: 2093-758X ).

Since being indexed in Scopus we have seen an increase in submissions: from 48 unsolicited manuscripts in 2021 to 63 in 2022. Now with triple crown status, I hope potential authors may consider KJWHN, especially those from a wider range of countries. High-quality studies that fit our aims and scope will be welcomed from authors over the world.

We have continued to publish English manuscripts at a steady rate, which will be meaningful for international readers to access our published content. Our collaborative work with Statistics Korea also continued in 2022, resulting in a statistics paper [3].

## Journal metrics

Data on manuscripts submitted to KJWHN this year, as of December 8, 2022 (Table 1), appear to reflect the power of being indexed in an international database. With a higher influx of submissions, the number of non-accepted manuscripts has also increased. Meanwhile, the acceptance rate has stabilized; a great improvement from 78.9% in 2019 and 63.8% in 2021, coming down to 48.4% in 2022.

## Future plans

Building on our hard work over the past few years since our initial MEDLINE application in 2020, KJWHN will be reapplying for MEDLINE inclusion shortly. We have made significant strides in meeting international guidelines. Preparing for Scopus, PMC, and ESCI indexing has helped us refine our policy and have a greater sensitivity toward communicating with readers. I wish to alert potential authors that we will strengthen our current policy on the following points.

### Clinical trial registration

Authors are expected to state the clinical registration number for interventional studies at submission, for example, Clinical Research Information Service (CRiS, https://cris.nih.go.kr) and ClinicalTrials.gov. Prospective registration is recommended and if retrospective registration was done, authors should also provide a brief description of the reason. An exception is interventional studies on students and/or healthcare providers that focus on knowledge, attitude, etc., that do not directly impact patient health outcomes.

### Data sharing

As noted in Table 1 of author guidelines (https://www.kjwhn.org/authors/authors.php), all interventional studies submitted after July 1, 2018 should include a statement on data sharing. Data sharing provides transparency and reproducibility of research so we strongly encourage authors to share deidentified data.

Furthermore, we have made efforts to overcome the weaknesses outlined in the review document notifying us of not being recommended for MEDLINE. The following describes our response to some major points:

*Point 1. High acceptance rate:* Although our acceptance rate when we initially applied for MEDLINE was relatively high (79% in 2019), this rate has stabilized over the years with probable booster effects since becoming indexed in an international database, as noted above. In 2022 we have reached less than 50% acceptance rate (Table 1) and will continue to seek to maintain this level. Rather than discouraging potential authors, we hope this will assure them that KJWHN is committed to publishing quality studies for research and practice.

*Point 2. Most articles are from Korean authors and written in Korean:* As stated in our aims and scope (https://kjwhn.org/about/index.php), our regional focus is mainly Korea, but we welcome submissions from all over the world. KJWHN still mainly receives submissions from domestic authors, but over the years, we have increased the proportion of English manuscripts to make these studies available to a wider audience. We welcome submissions from international authors, especially studies reporting on women’s health issues in Asia and/or using sex and gender-based analysis. And now, with triple crown indexing status, we expect to see more English manuscript submissions in the future. KJWHN is currently considering waivers and/or discounts of article processing charges for authors from developing countries, as a step to encourage more submissions from abroad.

*Point 3. Significant prevalence of descriptive correlational studies with no theoretical framework for why variables were chosen:* This has also been noted as a prevailing issue in other disciplines and journals [4,5]. KJWHN has been promoting specifying the theoretical framework through our annual workshops and communicating with authors during the review process to help them be more aware of the importance of stating their theoretical underpinnings. Potential authors are encouraged to include a description of their theoretical framework [6,7] and may refer to practical suggestions in the literature [4,8]. As for more variety in study designs, many society members usually choose to submit experimental study papers to journals with high impact factors. KJWHN has been advertising more actively that manuscripts on interventional studies, integrative reviews/scoping reviews, and systematic reviews and/or meta-analyses are particularly welcome. Over the past 3 years, we have published eight systematic reviews, one integrative review, two randomized controlled trials, and three quasi-experimental studies. We will continue to actively advertise and seek high-quality manuscripts on various study designs. KJWHN is planning a special issue on “Digital era education for women’s health and wellbeing” for September 2023 (https://www.kjwhn.org/file/kjwhn_call_for_paper.pdf). We welcome submissions covering the design, development, evaluation, and use of digital solutions that support the health and wellbeing of women through education.

## Appreciation for 2022 reviewers

KJWHN is indebted to our tireless reviewers, and our appreciation is extended to the following colleagues for their professional service this year:

Ahn, Suk Hee (Chungnam National University)

Chae, Hyun Ju (Joongbu University)

Cheon, Suk Hee (Sangji University)

Choi, Mi Young (National Evidence-based Healthcare Collaboration Agency)

Choi, So Young (Gyeongsang National University)

Chung, Chae Weon (Seoul National University)

Chung, Mi Young (SunMoon University)

Drake, Emily E. (University of Virginia)

Ha, Ju Young (Pusan National University)

Jeong, Geum Hee (Hallym University)

Jo, Myung Ju (The Catholic University of Korea)

Kang, Saemi (Gyeongsang National University)

Kang, Sookjung (Ewha Womans University)

Kim, Haewon (Seoul National University)

Kim, Hee Kyung (Kongju National University)

Kim, Hye Young (Keimyung University)

Kim, Hyun Kyoung (Kongju National University)

Kim, Jeung-Im (Soonchunhyang University)

Kim, Joungyoun (University of Seoul)

Kim, Kwang Ok (Dongju College)

Kim, Kyungah (Incheon Catholic University)

Kim, Mi Jong (Hannam University)

Kim, Mi Young (Woosuk University)

Kim, Myoung Hee (Semyung University)

Kim, Su Hyun (Nambu University)

Kim, Sun Ho (Chungbuk National University)

Kim, Yeunmi (Suwon Women University)

Kim, Young Man (Jeonbuk National University)

Kim, YoungJu (Daejeon Health Institute of Technology)

Kim, Yun Mi (Eulji University)

Ko, Eun (Sunchon National University)

Lee, Kyoung-Eun (Texas A & M University)

Lee, SunHee (Gimcheon University)

Moon, So Hyun (Chosun University)

Nho, Ju Hee (Jeonbuk National University)

Park, Mee Ra (Changshin University)

Park, Seo A (Gyeongbuk College of Health)

Park, So Mi (Yonsei University Mirae)

Seo, Minjeong (Gyeongsang National University)

Shin, Gi Soo (Chung-Ang University)

Son, Hae Kyoung (Eulji University)

Sung, Mi Hae (Inje University)

Yoo, Hana (Daejeon University)

Yoon, Ji Won (Shinhan University)

We especially wish to acknowledge Professors Eun Ko (Sunchon National University), Geum Hee Jeong (Hallym University), and Mi Hae Sung (Inje University) as “Reviewer of the Year 2022” and Professors Sukhee Ahn (Chungnam National University) and Joungyoun Kim (University of Seoul) for their statistical expertise.

The editorial board will continue to strive for excellence so that KJWHN will be able to serve its purpose of improving women’s health in Korea and beyond.

## Figures and Tables

**Table 1.** Basic statistics on manuscripts submitted to the *Korean Journal of Women Health Nursing* in 2022

Category	Data	Notes
Commissioned manuscripts (n)	8	4 *Editorials*, 4 *Issues & Perspectives*
Unsolicited manuscripts (n)	63	
Accepted manuscripts (n)	30	
Non-accepted manuscripts (n)	27	19 Rejected, 7 rejected before review (unsuitable), 1 withdrawn
Manuscripts reviewed and determined (n)	49	30 accepted, 19 rejected
Manuscripts under review or revision (n)	13	
Acceptance rate (%)	48.4	30/62=0.484
Average time from submission to acceptance (day)	56.4	
